# First detection of F1534C knockdown resistance mutation in *Aedes aegypti* (Diptera: Culicidae) from Cameroon

**DOI:** 10.1186/s40249-020-00769-1

**Published:** 2020-11-02

**Authors:** Aurelie P. Yougang, Basile Kamgang, Theodel A. Wilson Bahun, Armel N. Tedjou, Daniel Nguiffo-Nguete, Flobert Njiokou, Charles S. Wondji

**Affiliations:** 1 Centre for Research in Infectious Diseases, P.O. Box 13591, Yaoundé, Cameroon; 2 Parasitology and Ecology Laboratory, Department of Animal Biology and Physiology, Faculty of Science, University of Yaoundé 1, P.O. Box 812, Yaoundé, Cameroon; 3 Laboratory of Vertebrate and Invertebrate Bioecology, Faculty of Science and Technology, Marien-Ngouabi University, Brazzaville, Congo; 4 Laboratory of Biology and Applied Ecology, Department of Animal Biology, Faculty of Science, University of Dschang, P.O. Box 96, Dschang, Cameroon; 5 Liverpool School of Tropical Medicine, Pembroke place, Liverpool, L3 5QA UK

**Keywords:** *Aedes aegypti*, Insecticide resistance, *Kdr* mutation, Cameroon, Arbovirus

## Abstract

**Background:**

*Aedes* borne viral diseases, notably dengue, are increasingly reported in Cameroon with *Aedes aegypti* being a major vector. Data on insecticide resistance of this vector and underlying mechanisms needed for outbreak preparedness remain scarce in Cameroon. Here, we present the nationwide distribution of insecticide resistance in *Ae. aegypti* and investigate the potential resistance mechanisms involved.

**Methods:**

Immature stages of *Ae. aegypti* were collected between March and July 2017 in 13 locations across Cameroon and reared until G1/G2/G3 generation. Larval, adult bioassays, and piperonyl butoxide (PBO) synergist assays were carried out according to World Health Organization guidelines. F1534C mutation was genotyped using allele specific polymerase chain reaction in field collected adults (Go) and the polymorphism of the sodium channel gene was assessed. The *χ*^2^ test was used to compare the mortality rate between bioassays with insecticides only and bioassays after preexposure to PBO synergist.

**Results:**

Larval bioassay revealed that all the three populations tested with temephos were susceptible. Adult bioassays showed a good level of susceptibility toward both pyrethroids tested, 0.25% permethrin and 0.05% deltamethrin, with six out of 10 populations susceptible. However, two populations (Douala and Edéa) were resistant (deltamethrin [73.2–92.5% mortality], permethrin [2.6–76.3% mortality]). The resistance to 4% dichlorodiphenyltrichloroethane was observed in four out of 10 populations tested (16.8–87.1% mortality). Resistance was also reported to carbamates including 0.1% propoxur (60.8–87.1% mortality) and to 0.1% bendiocarb (82.9% mortality). All populations tested were fully susceptible to 1% fenitrothion. A partial recovery of susceptibility was observed in the pyrethroid resistant population of Douala after pre-exposed to PBO suggesting the implication of cytochrome P450 monoxygenases permethrin resistance. Genotyping and sequencing detected the F1534C *kdr* mutation in the two pyrethroid resistant locations of Edéa and Douala, with allelic frequency of 3.3% and 33.3% respectively. However, the high genetic diversity of the sodium channel gene supports the recent introduction of this mutation in Cameroon.

**Conclusions:**

This study revealed the contrasting resistance profiles to insecticides of *Ae. aegypti* populations in Cameroon suggesting that, instead of a unique nationwide control approach, a regionally adapted strategy will be needed to control this vector. The localised distribution of the F1534C *kdr* mutation supports this region-specific control strategy.

## Background

The mosquito *Aedes aegypti* Linneaus, 1762 (Diptera: Culicidae) is the main vector of several arboviral related diseases such as dengue, Zika, chikungunya, and yellow fever in subtropical and tropical world. This domestic mosquito usually bites during daylight, feeding mainly on humans, mating and resting indoor/outdoor, and breeding in man-made containers in and around human habitations [1].

In Cameroon, where several cases of arboviral related diseases such as dengue [2–5], chikungunya [6, 7], yellow fever [8] and Zika [9] are increasingly reported, it was demonstrated that *Ae. aegypti* is present across the country and found as dominant *Aedes* species in some locations notably in the northern part [10]. It was also demonstrated that local *Ae. aegypti* populations are able to transmit dengue [11], Zika [12] and yellow fever [13] viruses in different urban settings in Cameroon. Prevention of large outbreaks caused by these virus relies on control of *Aedes* vectors based on destruction of breeding sites and insecticide-based interventions such as treatment of breeding sites with larvicides, insecticide-treated nets [14] and space spraying of adulticides in emergency situations [15, 16]. However, at the operational level, many vector control programmes are facing the challenge of the development of insecticide resistance in *Ae. aegypti.* Indeed, *Ae. aegypti* has been found to be resistant to several classes of insecticides in different regions across the world with significant variation according to the population’s origin and the insecticide classes [17–24].

The insecticide resistance in mosquitoes is primarily associated to two main mechanisms: insensitivity of target sites (target-site resistance) due to mutations that reduce the binding affinity between the insecticide and the target site, and metabolic resistance resulting in an overproduction of enzymes that will facilitate the detoxification of insecticides [25, 26]. The metabolic resistance through overexpression of detoxification genes is a common resistance mechanism in *Ae. aegypti* as well as in *Ae. albopictus*. The three main enzyme families responsible for insecticide resistance in mosquitoes are the monooxygenases (cytochrome P450s), glutathione S-transferases (*GSTs*), and carboxylesterases (*COEs*) [26, 27].

Target site resistance is caused by mutations in target genes such as the acetylcholinesterase (*Ace-1*), the *GABA* receptor and the voltage-gated sodium channel (*VGSC*) causing knockdown resistance (*kdr*). One of the most important target site resistance for mosquitoes is *kdr* as it confers resistance to both pyrethroids and dichlorodiphenyltrichloroethane (DDT). Eleven *kdr* mutations in *VGSC* domain I-IV have been identified in *Ae. aegypti* around the world and the association between F1534C, V1016G, I1011M, and V410L mutations and pyrethroid resistance has been established [22, 28, 29]. In Africa 1534 and 1016 mutations have been previously reported in *Ae. aegypti* in Burkina-Faso and Ghana [21], and 410 mutation in Angola [30]. In Cameroon, data on insecticide resistance in *Ae. aegypti* and resistance mechanisms involved are very limited apart from the preliminary studies highlighting the resistance of this species to DDT, deltamethrin, and bendiocarb in some locations and suggesting the implication of cytochrome P450 enzymes in pyrethroids and DDT resistance [19, 20]. Thereby, we present here the nationwide distribution of insecticide resistance to *Ae. aegypti* and investigate the potential implication of 1534 *kdr* mutation in the pyrethroid resistance. This was done by assessing the presence and distribution of the 1534C resistant allele and analysing the genetic diversity of the related portion of the sodium channel gene country-wide.

## Methods

### Collection of mosquitoes

**Fig. 1:**
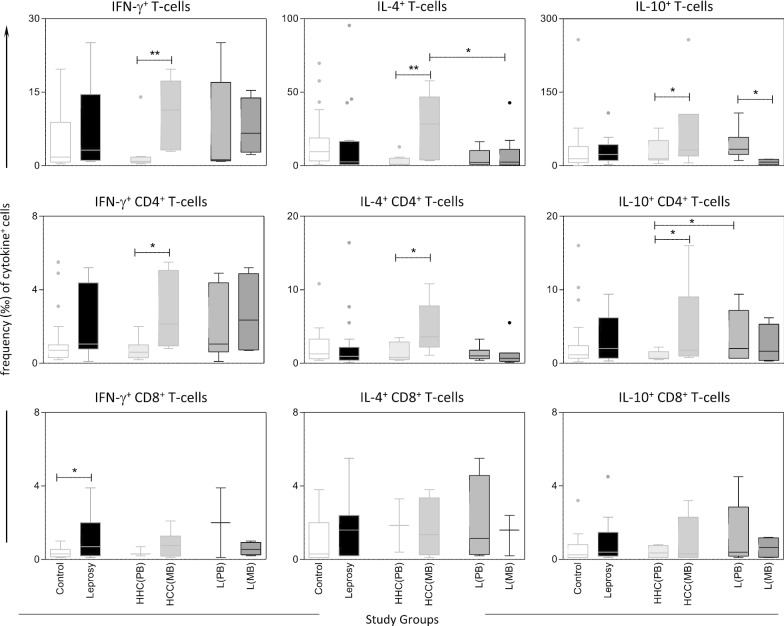
Map of Cameroon showing the sampling sites

Immature stages of *Aedes* were collected between March and July 2017 in 13 locations across Cameroon (Fig. 1): Maroua (10° 35′ N; 14° 18′ E), Benoué national park (08° 20′ N; 13° 50′ E), Garoua (09° 18′ N; 13° 24′ E), Mbé (07° 51′ N; 13° 35′ E), Banyo (06° 45′ N; 11° 49′ E), Tibati (06°28′N; 12°38′E), Meiganga (06°31′N; 14°18′E), Ngaoundéré (07° 19′ N; 13° 35′ E), Edéa (03° 48′ N; 10° 08′ E), Limbé (04° 00′ N; 09° 13′ E), Douala (04° 03′ N; 09° 42′ E), Melong (05° 07′ N; 09° 57′ E), and Yaoundé (03° 52′ N; 11° 31′ E). Detailed characteristics of each collection site are presented in previous studies [10]. In each location, mosquitoes were collected in peri-urban and downtown at a minimum of 20 positive larval breeding places per site. Larvae/pupae of *Aedes* mosquitoes were transported to an insectary and pooled together according to the city and maintained until they emerged as adults before morphological identification using a suitable taxonomic key [31, 32]. Adult mosquitoes were maintained at insectary and reared in the controlled conditions (27 °C ± 2 °C; relative humidity 80% ± 10%). Mosquitoes identified as *Ae. aegypti* were reared until generation until G1/G2/G3. *Ae. aegypti* New Orleans (NO) strain was used as reference susceptible strain.

### Insecticides susceptibility tests

#### Larval bioassays

Larval tests were conducted following World Health Organization (WHO) protocol [33]. The late third- and early fourth instar larvae were used for each mosquito population. Four replicates were tested with 20–25 larvae per replicate and per concentration. The susceptibility of larvae was evaluated against technical-grade temephos (97.3%; Sigma Aldrich-Pestanal, Seelze, Germany). First, stock solutions and serial dilution were prepared in 95% ethanol for temephos and stored at 4 °C. Seven concentrations ranging from 0.0005 and 0.006 mg/L have been used to test both field strain and susceptible lab strain (New Orleans). Larvae were not fed during the experiment and the conditions of the room were 27 ± 2 °C (temperature) and 70 ± 10% (relative humidity). Larval mortality was recorded after 24 h of exposure to larvicide.

All the results were analyzed with win DL software (v. 2.0, CIRAD-CD, Montpellier, France) to determine the lethal concentration for 50% (LC_50_) and 95% (LC_95_) of the populations. Resistance ratios (RR_50_ and RR_95_) were calculated using LC_50_ and LC_95_ rates from *Ae. aegypti* field populations compared with the LC_50_ and LC_95_ rates of the reference strain. The resistance levels were ranked into three categories: low resistance (RR_50_ < 5), medium or moderate resistance (5 ≤ RR_50_ ≤ 10), and high resistance (RR_50_ > 10) [33].

#### Insecticides susceptibility bioassays

Adult bioassays were carried out according to WHO guidelines [33]. Six insecticides were tested: 0.25% permethrin (Type I pyrethroid), 0.05% deltamethrin (Type II pyrethroid), 4% DDT (organochlorine), 0.1% propoxur (carbamate), 0.1% bendiocarb (Carbamate), and 1% fenitrothion (organophosphate). Four replicates of 20–25 unfed two to five days old female *Ae. aegypti* were exposed to insecticide-impregnated papers for 1 h under the insectary conditions described above, and then transferred to holding tubes with access to 10% sugar solution. The mortality rate was recorded 24 h later. The dead mosquitoes were stored in silica gel desiccant and the survivor in RNA later at -80 °C freezer. The resistance status was defined as follows: susceptible (mortality rate between 98 and 100%), probable resistance (mortality rate between 90 and 98%) and resistant (mortality rate inferior to 90%) [33].

#### Synergist assays

In order to investigate the potential role of oxidases in the metabolic resistance mechanism, synergist assay was performed in Douala population using 4% piperonyl butoxide (PBO). 2–5-day-old adults were pre-exposed for one hour to PBO-impregnated papers and immediately exposed to permethrin. Mortality was scored 24 h later and compared to the results obtained with permethrin without synergist according to the WHO standard [34]

### F1534C *kdr* genotyping using allele specific polymerase chain reaction (PCR)

As previous study in Central Africa [18] had not reported any mutation associated to pyrethroid resistance, we decided to focus our analysis in F1534C mutation which is mostly found worldwide including in West Africa [21, 35]. For this purpose, genomic DNA of 30 individual mosquitoes per populations was extracted using Livak protocol [36]. That DNA was used to genotype the F1534C mutation which has been described to be associated to pyrethroids and DDT resistance. Allele specific PCR assays were performed following using Harris et al. protocol [37]. Each PCR reaction was performed in a 15 μl volume containing: 1 μl of DNA sample, 0.4 units of Kapa *Taq* DNA polymerase, 0.12 μl of 25 mmol/L dNTPs (0.2 mmol/L), 0.75 μl of 25 mmol/L MgCl_2_ (1.5 mmol/L), 1.5 μl of 10 × PCR buffer (1 ×), 0.51 μl of each primers (0.34 mmol/L). The amplification consisted of 95 °C for a 5 min heat activation step, followed by 35 cycles of 94 °C for 30 s, 55 °C for 30 s and 72 °C for 45 s with a 10 min final extension step at 72 °C. The PCR products were separated on agarose gel 3% stained with Midori green.

### Polymorphism of the voltage-gated sodium channel (*VGSC*) gene

To assess the polymorphism of the *VGSC* gene and detect possible signatures of selection, a fragment of this gene spanning the F1534C mutation (a part of segment 6 of Domain III) was amplified and sequenced in 130 G_0_ field collected mosquitoes from 13 locations across Cameroon. PCR reactions were carried out using 10 pmol of each primer [aegSCF7 (GAGAACTCGCCGATGAACTT) and aegSCR7 (GACGACGAAATCGAACAGGT)] and 20 ng of genomic DNA as template in 15 μl reactions containing 1 × Kapa *Taq* buffer, 0.2 mmol/L dNTPs, 1.5 mmol/L MgCl_2_, 1U Kapa *Taq* (Kapa biosystems) [38]. The cycle conditions were 94 °C for 3 min, 35 cycles of 94 °C for 15 s, 55 °C for 30 s, and 72 °C for 30 s, followed by a final elongation step at 72 °C for 10 min. Amplicons from the PCR were analysed by agarose gel electrophoresis stained with Midori green and visualized under UV light. The amplified fragments of the expected size were purified using ExoSAP following manufacturer recommendations and directly sent for sequencing. The sequences were corrected with BioEdit software (v 7.1.8, London information retrieval ltd, London, UK) and aligned with Clustal W [39]. DNAsp (v 6.10.01, Universitat de Barcelona, Barcelona, Spain) [40] was used to define the haplotype phase and compute the genetic parameters including the number of haplotypes (h), the number of polymorphism sites (S), haplotype diversity (Hd), and nucleotide diversity (π). The statistical tests of Tajima [41] and Fu Fs [42] were estimated with DnaSP in order to establish non-neutral evolution and deviation from mutation-drift equilibrium. Different haplotypes obtained and reference sequences were used to construct the maximum likelihood phylogenetic tree using Mega 6.0 [43]. A haplotype network was built using TCS [44] and TcsBu [45] programs to further assess the genealogical relationship between haplotypes.

## Results

### Larval bioassays

Larval assays were tested with temephos for three populations due to the limited number of larvae (Table 1). Analysis revealed that the resistance ratio for all populations tested was less than 2 suggesting the susceptibility of these populations to temephos.

**Table 1:** Larval bioassays with temephos against *Aedes aegypti* larvae

Strain and Site	*n*	LC_95_ (mg/L) (95% *CI*)	RR_95_	LC_50_ (mg/L) (95% *CI*)	RR_50_
NO lab strain	531	0.0046 (0.0042–0.0051)	–	0.0026 (0.0025–0.0028)	–
Edéa	531	0.0046 (0.0036–0.0094)	1.00	0.0021 (0.0007–0.0028)	0.80
Douala	483	0.0078 (0.0069–0.0092)	1.68	0.0039 (0.0037–0. 0042)	1.47
Yaoundé	537	0.0034 (0.00258–0.0069)	0.74	0.0015 (0.0009–0.0020)	0.59

*n* number of larvae tested; LC_95_ and LC_50_ 95 and 50% lethal concentrations; *CI* Confidence interval; RR resistance ratio; NO New Orleans

### Insecticide resistance profile in adults

**Fig. 2:**
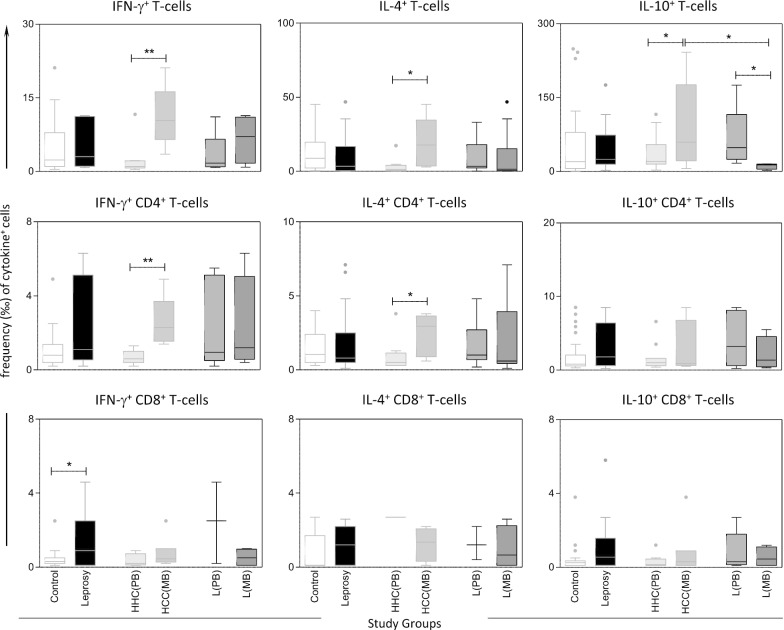
Mortality rates of adult *Aedes aegypti* from 10 locations in Cameroon 24 h after exposure to insecticides alone or with 1 h pre-exposure to synergist. Error bars represent standard error of the mean. *DDT* Dichlorodiphenyltrichloroethane, *PBO* Piperonyl butoxide. a, Douala; b, Limbe; c, Edéa; d, Parc Benoué; e, Ngaoundéré; f, Maroua; g, Banyo; h, Tibati; i, Meiganga; j, Melong

**Fig. 3:**
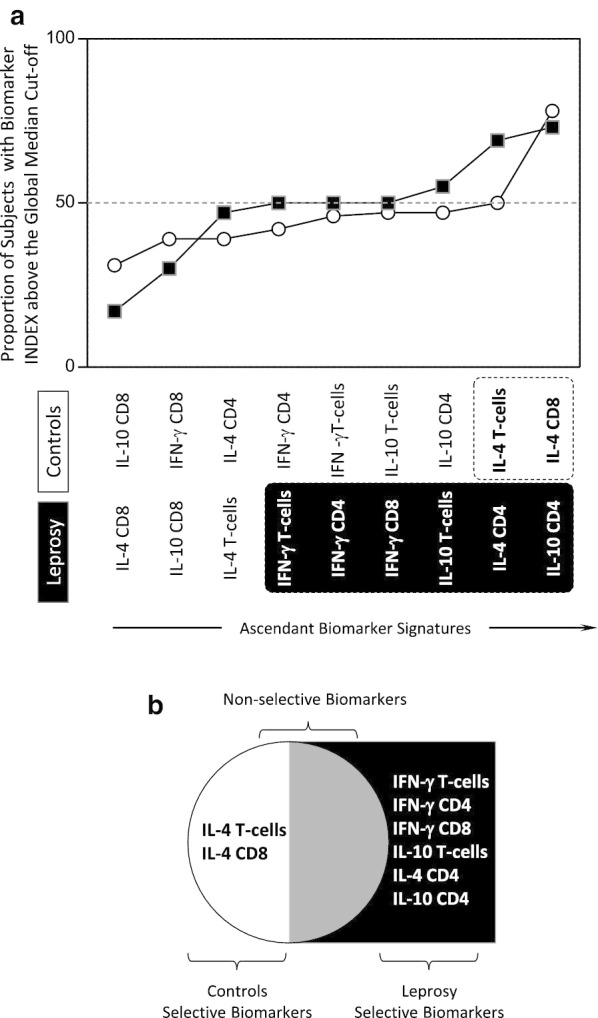
Map showing the insecticide resistance status of *Aedes aegypti* in Cameroon. DDT: Dichlorodiphenyltrichloroethane

Bioassays were performed in 10 *Ae. aegypti* populations collected across Cameroon (Figs. 2 and 3). Analysis revealed that four populations out of 10 were resistant to DDT with mortality rate ranging from 16.8% in Douala to 77.3% in Ngaoundéré populations. Six other populations were either probable resistant or susceptible with percentage of mortality varying from 92.32% in Tibati to 100% in Maroua. A good susceptibility level was observed against both pyrethroids tested, type I pyrethroid permethrin and type II pyrethroid deltamethrin, with six susceptible populations, two probable resistant and two resistant populations with lowest mortality rate (2.56%) in Douala population for permethrin (Fig. 2). A moderate level of resistance was reported against carbamates notably to propoxur for which mortality rates between of 60.82% and 87.71% in Edéa and in Meiganga populations, respectively. Nevertheless, probable resistance was detected in four populations with mortality rate ranging from 95.65% in Tibati to 97.33% in Melong. Only one population (Douala) was fully susceptible to propoxur. The unique population from Limbé tested to bendiocarb was resistant with mortality rate of 82.95%. All populations tested across Cameroon exhibited a full susceptibility toward the organophosphate fenitrothion which is in line with temephos susceptibility observed in larvae (Fig. 3).

### Synergist assay with PBO

The synergist assay analysis revealed a partial recovery of susceptibility to permethrin after PBO pre-exposure (Fig. 2) to Douala population (2.56 ± 1.48% without PBO vs 14.28 ± 5.8% with PBO, *P* < 0.005) suggesting that the cytochrome P450 monooxygenases are also playing a role in pyrethroids resistance in this populaton.

### F1534C *kdr* genotyping using allele specific PCR

A total of 331 specimens of *Ae. aegypti* from 13 locations across Cameroon was successfully amplified (Table 2). Among them, 320 (96.68%) were susceptible (1534 F/F), 8 (2.41%) were heterozygote resistant (1534 F/C), and 3 (0.91%) were homozygote resistant (1534 C/C). Overall, allelic frequency of susceptible was 0.98 while for resistant was 0.02. The F1534C mutation was found in only two populations: Edéa and Douala with allelic frequencies of 3.33% and 33.33% respectively (Table 2).

**Table 2:** F1534C genotype numbers and the allelic frequency of the C mutation of *Aedes aegypti*

Location	F1534 genotypes			FF + FC + CC	Allelic frequencies	
	FF	FC	CC		% F	% C
Benoué national park	24	0	0	24	100	0
Maroua	27	0	0	27	100	0
Garoua	30	0	0	30	100	0
Mbé	17	0	0	17	100	0
Ngaoundéré	25	0	0	25	100	0
Banyo	29	0	0	29	100	0
Tibati	26	0	0	26	100	0
Meiganga	24	0	0	24	100	0
Edéa	29	0	1	30	96.67	3.33
Limbé	27	0	0	27	100	0
Douala	8	8	2	18	66.67	33.33
Melong	24	0	0	24	100	0
Yaoundé	30	0	0	30	100	0
Total	320	8	3	331	97.89	2.11

F: phenylalanine; C: cysteine; F/F: absence of the F1534C mutation; F/C: presence of the F1534C mutation with 2 alleles: one resistant, allele C and another susceptible F allele; C/C: presence of the F1534C mutation with the 2 resistant alleles

### Genetic diversity of *VGSC* in *Ae. aegypti*

**Fig. 4:**
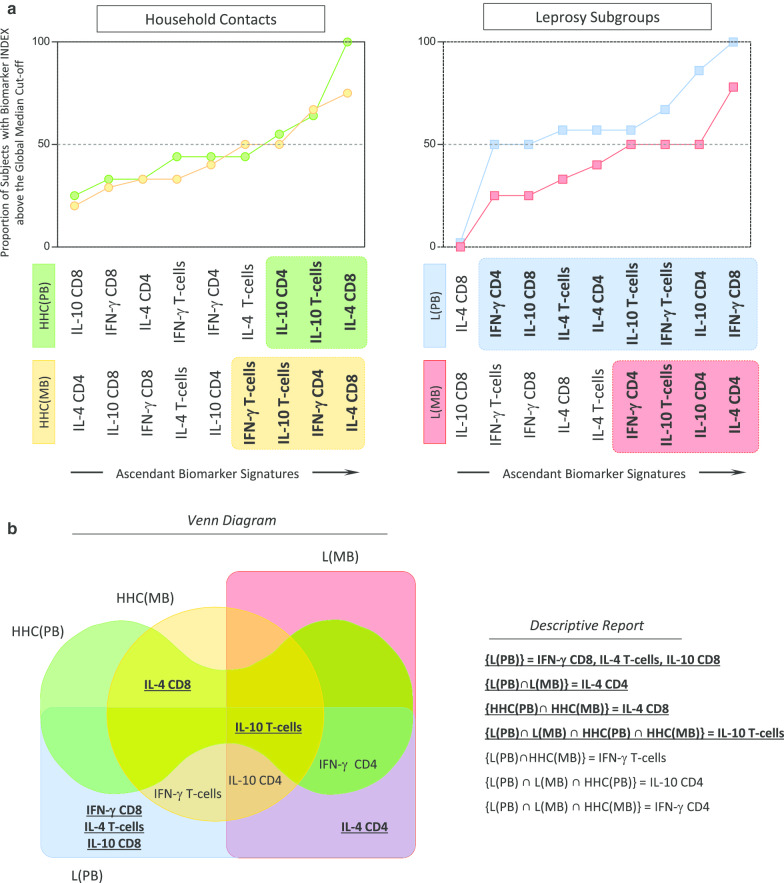
3: Sequencing of the portion of the voltage gated sodium channel gene spanning the F1534C mutation. **a** Sequence alignment of the voltage gated sodium channel gene at the F1534C point mutation in field collected adult mosquitoes (F0), **b** Chromatogram traces showing the three genotypes at the 1534 coding position

**Fig. 5:**
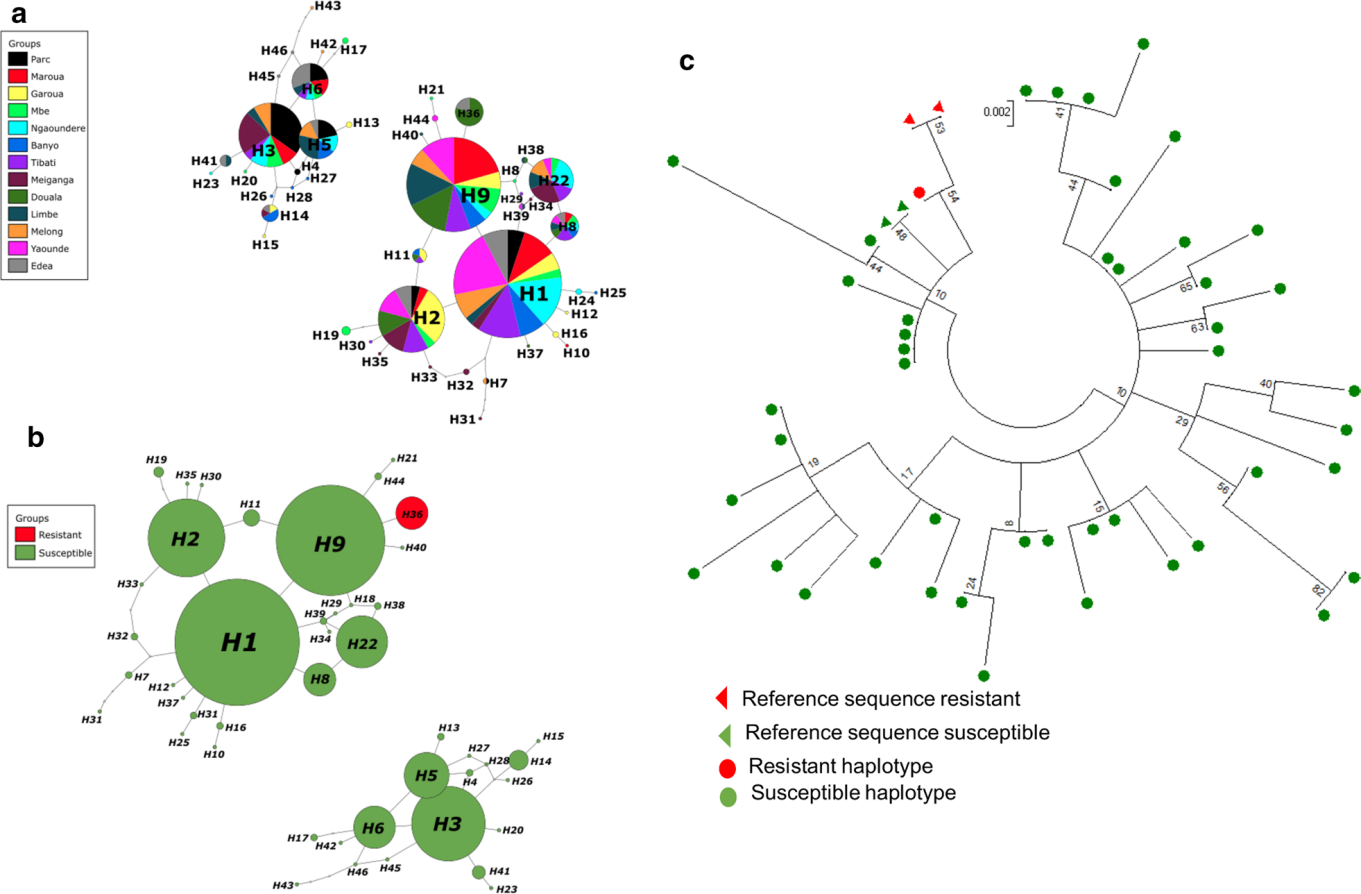
Pattern of genetic variability and polymorphism of the voltage-gated sodium channel in *Aedes aegypti*. **a** Haplotype network for the *VGSC* sequence taking into account different populations; **b** Haplotype network for the *VGSC* sequences taking into account the resistance status; **c)** Phylogenetic tree of *VGSC* DNA sequences (fragment) by maximum-likelihood with Kimura model

One hundred and twenty-two field collected *Ae. aegypti* from 13 locations were successfully sequenced for a 201 bp fragment of the *VGSC* gene spanning the codon 1534. Analysis confirmed the presence of mutation 1534C in Douala and Edéa samples (Fig. 4). Overall, 25 polymorphic sites, 38 haplotypes (46 haplotypes, when taking into account insertions or deletions) with a high haplotype diversity (0.879) and low nucleotide diversity (0.010) (Table 3). Among these haplotypes, H1 (15.98%), H10 (13.93%), H2 (9.83%) and H3 (9.42%) were the most represented (Fig. 5a). The resistant haplotype H36 was detected in Douala (80%) and Edéa (20%) populations (Fig. 5a, b). A maximum likelihood (ML) tree of the sequences analysed confirms a high diversity with the probable four clusters (Fig. 5c). Globally, all the statistics estimated were negatives (D = − 1.479, Fu’s Fs = − 33.498) with Fu’s Fs statistically significant (Table 3). Negative values for these indexes indicate an excess of rare polymorphisms in a population and suggest a recent expansion of the gene studied across the populations or background selection.

**Table 3:** Genetic diversity parameters of F1534C mutation among Cameroonian *Aedes aegypti* populations

Populations	2N	S	Syn	Nsyn	π	H	Hd	D	Fu Fs
Benoué national park	20	5	4	1	0.006	6	0.726	− 0.591	− 1.874
Maroua	18	5	4	1	0.006	5	0.745	− 0.703	− 0.945
Garoua	20	9	7	2	0.011	9	0.863	− 0.592	− 3.113*
Mbe	18	9	8	1	0.014	9	0.922	0.023	− 2.581*
Ngaoundéré	20	6	6	0	0.007	6	0.763	− 0.479	− 1.195
Banyo	16	7	5	2	0.012	9	0.908	0.318	− 3.584*
Tibati	20	5	4	1	0.008	8	0.868	0.379	− 3.062*
Meiganga	20	12	11	1	0.013	9	0.847	− 1.020	− 2.544*
Douala	20	6	4	2	0.008	7	0.784	− 0.122	− 1.850
Limbé	20	5	4	1	0.009	8	0.853	0.836	− 2.590*
Melong	14	7	7	0	0.011	6	0.813	− 0.097	− 0.787
Yaoundé	20	5	4	1	0.006	7	0.800	− 0.283	− 2.653*
Edéa	18	8	6	2	0.009	9	0.895	− 0.740	− 4.088*
Total	244	25	18	8	0.010	38	0.879	− 1.479	33.498***

2N, number of sequences; S, number of polymorphic sites; h, number of haplotypes; Hd, haplotype diversity; π, nucleotide diversity; Syn and Nsyn, synonymous and non-synonymous mutation; D and Fs, Tajima’s D and Fu Fs statistics, *degree of significance

## Discussion

This study investigated the nationwide insecticide profile of *Ae. aegypti* in Cameroon and assessed the contribution of F1534C *kdr* mutation in insecticide resistance. Larval bioassays analysis revealed that all *Ae. aegypti* populations tested were susceptible to the organophosphate temephos. This observation is similar to those made by previous studies from several countries in Central Africa such as in Cameroon [19], Central African Republic [18], and Republic of the Congo [24]. Nonetheless, the resistance of *Ae*. *aegypti* to this compound was reported in several countries such as in Laos [23], Brazil [46], Thailand [47], Malaysia [48], and Cape Verde [49]. This organophosphate is the larvicide mainly used to control *Aedes* larvae by treating water storage containers [46, 50, 51]. However, selection of the resistance results from extensive and long-term use of the product incriminated, whereas in our knowledge, temephos had never been used in vector control programs in Cameroon. This probably explains the full susceptibility reported in *Ae. aegypti* as suggested previously [19].

Adult bioassays analysis revealed that four *Ae. aegypti* samples were found resistant to DDT and the remaining were either susceptible or probably resistant. A decreasing susceptibility of the *Ae. aegypti* population from Yaoundé and Brazzaville towards DDT was already mentioned in 1970s [52], suggesting that this resistance may have resulted from a continuing selection pressure on *Aedes* populations as suggested previously [18, 19]. Indeed, recent study in Central Africa reported the resistance of *Ae. aegypti* to DDT [18, 20, 24]. The full susceptibility reported in some populations such as in Maroua and Meiganga shows that the DDT resistance is not nationwide in Cameroon and suggests that this compound can still be effective to control *Ae. aegypti*.

*Aedes aegypti* populations showed a good level of susceptibility toward type I pyrethroid permethrin with only three resistant populations out of 10 tested. The loss of susceptibility to this pyrethroid was previously reported in Cameroon [20] and outside Africa [23, 48]. Similarly, a good level susceptibility was reported against type II pyrethroid deltamethrin. These results suggest that the resistance to deltamethrin and permethrin has not yet spread country-wide and these insecticides are still effective to control *Aedes* in some locations of Cameroon. A loss of sensitivity was observed to carbamates notably propoxur with moderate level of resistance in some locations such as: Limbé, Edéa, and Tibati. This result is comparable to previous reports in the Republic of the Congo [24] and in Burkina Faso [29] in Africa and in several countries outside Africa such as Malaysia [48], Pakistan [53], and Saudi Arabia [54]. The source of selection driving the observed resistance to DDT, permethrin, deltamethrin, propoxur and bendiocarb in some *Ae. aegypti* populations remains unclear notably as the use of insecticides against *Aedes* is limited in the region [19, 20]. As suggested previously [18, 20], domestic used of insecticides through the indoor spraying and impregnating bed nets, and agriculture use could be the main source of resistance selection in *Aedes* vectors in Central Africa. Indeed, the use of pesticides in agriculture for the protection of market gardening could also promoted the emergence of resistance in mosquitoes by contamination of breeding sites and resting places of mosquitoes [55].

A partial recovery of susceptibility to permethrin was reported in Douala population after pre-exposure to PBO synergist. This result indicates that the cytochrome P450 monooxygenases are playing a role in the observed resistance perhaps in association with other enzyme families or/and other resistance mechanisms as the recovery was only modest. The implication of cytochrome P450 monooxygenases in *Ae. aegypti* resistance has been previously reported in several regions in the world including Central Africa the sub-region [18, 20].

The F1534C mutation is common in *Ae. aegypti* and has a worldwide distribution [22] although it was not yet detected in Cameroon [20]. Our analysis revealed the first evidence of this mutation in *Ae. aegypti* from two locations (Edéa and Douala) of Cameroon. This *kdr* mutation was previously reported in Africa in Ghana [21] and Burkina Faso [29, 35]. The allelic frequency of this mutation observed in Cameroon (3.3–33.3%) is low compared to those found in Ghana for example (33.3–68.42%) [21]. In fact, the result of neutrality test suggests a recent selection of this mutation in Cameroon with a potential origin from Douala. The presence of a unique resistant haplotype H36 support a unique origin of the 1534C allele in Cameroon probably in Douala with a gradual spread in the country. As Douala is the main port of Cameroon, it is not excluded that the 1534C may have been imported recently instead of a de novo local selection. Indeed, our sequences clustered with the reference sequences downloaded in GenBank (accession numbers: MF794989.1, MF794985.1 and MF794990.1) coming from Thailand [56].

The absence of a reduced diversity at the *VGSC* in Douala and Edéa as shown from ML tree and TCS haplotype network is due to recent selection of this resistant allele in these locations which is further supported by the low frequency of homozygote CC. However, these populations will need to be monitored as increasing pressure may lead to the further selection associated with reduced diversity as seen in other locations such as in Malaysia [48] for *Ae. aegypti*. Such increasing selection pressure on mosquitoes populations have also been observed for metabolic resistance genes such as *GSTe2* for the L119F in Benin [57] or cytochrome P450 (*CYP6P9a/b*) [58–60] leading to drastic reduced diversity.

In addition, it will be interesting to genotype other mutations such as 1016 and 410 which have been found implicated in *kdr* resistance in *Ae. aegypti* [22, 28–30] and investigate the genes involved in metabolic resistance such as *CYP9* overexpressed in several regions worldwide including in Africa [35].

## Conclusions

Our result revealed a variable level of susceptibility among populations towards insecticides tested across the country. The full susceptibility to organophosphates at both larval (temephos) and adult stages (fenitrothion) makes this insecticide class very suitable for control *Ae. aegypti* in Cameroon. Furthermore, this study revealed for the first time the presence of target site mutation F1534C in Cameroon. Data generated in this study could serve as baseline to implement further investigations and planning efficient insecticide-based interventions against *Ae. aegypti* in Cameroon.

## Data Availability

All the relevant data generated during this study are included in the manuscript.

## Abbreviations

AS: Allele specific
DDT: Dichlorodiphenyltrichloroethane
DNA: Deoxyribonucleic acid
kdr: Knockdown resistance
LC: Lethal concentration
PBO: Piperonyl butoxide
PCR: Polymerase chain reaction
RR: Resistance ratio
VCRU: Vector control research unit
*VGSC*: Voltage-gated sodium channel
WHO: World Health Organization
